# An Apology

**Published:** 1895-06

**Authors:** 


### HOMŒOPATHIC PHYSICIAN,

A MONTHLY JOURNAL OF

HOMŒOPATHIC MATERIA MEDICA AND CLINICAL MEDICINE.

"If our school ever gives up the strict inductive method of Hahnemann, we are lost, and deserve only to be mentioned as a caricature in the history of medicine."—CONSTANTINE HERING.

Vol. XV. JUNE, 1895. No. 6.

### EDITORIAL.

AN APOLOGY.—The editor regrets that owing to the crowded condition of his pages he has been obliged to postpone his notes on materia medica until next number. He also regrets the delay in the appearance of the journal last month and this month, which is owing to several very serious cases of sickness that have required his unremitting attention, and have thus prevented him from giving to the journal the amount of time it so justly merits. It is hoped that this delay may be remedied in a short time.

